# Overexpression of EMT-inducing transcription factors as a potential poor prognostic factor for hepatocellular carcinoma in Asian populations: A meta-analysis

**DOI:** 10.18632/oncotarget.18352

**Published:** 2017-06-02

**Authors:** Tao Wan, Tianwei Zhang, Xiaoying Si, Yanming Zhou

**Affiliations:** ^1^ Department of Hepatobiliary and Pancreatovascular Surgery, First Affiliated Hospital of Xiamen University, Xiamen, China

**Keywords:** EMT-TFs, hepatocellular carcinoma, biomarker, prognosis, meta-analysis

## Abstract

**Background and Objectives:**

The clinical relevance of epithelial to mesenchymal transition (EMT) in hepatocellular carcinoma (HCC) progression has been highlighted during the last decade. The zinc finger E-box binding homeobox (ZEB) family, the zinc-finger transcriptional repressor (SNAI) family, and the basic helix-loop-helix transcription factor (Twist) family, known as the prominent EMT-inducing transcription factors (EMT-TFs), played a crucial role in the process of EMT. Here, this meta-analysis aimed to evaluate the prognostic value of EMT-TFs high expression in patients with HCC after hepatectomy.

**Results:**

A total of 10 studies involving 1334 patients were retrieved for analysis, the synthetic date indicated that EMT-TFs overexpression was associated with poor postoperative overall survival (OS) [HR = 1.71; 95% CI: 1.40–2.08; *p* < 0.00001] in HCC. The subgroup analyses revealed that overexpression of each individual EMT-TF (in addition to ZEB2) tended to be associated with poor OS. Moreover, EMT-TFs overexpression correlated with TNM stage, poor histological differentiation, intrahepatic metastasis and vascular invasion.

**Materials and Methods:**

Relevant literature search in the PubMed, Web of Science database and Cochrane Library was performed to retrieve all eligible studies. The pooled hazard ratio (HR) or odds ratio (OR) with its 95% confidence interval (CI) were calculated to investigation clinicopathological and prognostic significance of EMT-TFs expression in HCC.

**Conclusions:**

EMT-TFs overexpression indicated an unfavorable prognosis in HCC patients following curative resection.

## INTRODUCTION

Hepatocellular carcinoma (HCC) is one of the most common malignant tumors and ranks the third highest cause of cancer-related deaths worldwide [1, 2]. The resection rate of HCC has increased over decades due to the improvements in early diagnostic methods and surgical techniques. However, the postoperative recurrence rate and overall survival (OS) are not optimistic due to limited response to various adjunctive therapies and aggressive behaviors in advanced stages of HCC [3]. Thus, an accurate understanding of the biological behavior of therioma is critical in predicting the prognosis of HCC patients. Traditional prognostic factors related to the clinicopathological characteristics of the neoplasm after hepatic resection such as tumor size, vascular invasion, tumor-node-metastasis(TNM) stage, functional liver reserve and Child-Pugh class have a limited clinical value for outcome prediction [4]. Therefore, a variety of other potential molecular predictive markers need to be further identified.

A sequential process, including escape from the primary tumor site, local invasion, systemic transport through vascular or lymphatic vessels, adhesion to distant organs, re-colonization, and expansion, is believed to be involved in hepatic carcinogenesis. Epithelial to mesenchymal transition (EMT) is known to play a pivotal role in the diffusion of cancer cells and the growth of tumors, in which epithelial cells lose their polarity and cell-cell contacts due to repressed expression of E-cadherin and up-regulated expression of mesenchymal markers such as N-cadherin, vimentin and α–smooth muscle actin (α–SMA) [5]. EMT could enhance not only the capacity of invasion and migration but resistance to apoptosis and chemoresistance in cancer. EMT may alter the gene expression of epithelial cells due to the activation of EMT-inducing transcription factors (EMT-TFs). In this meta-analysis, we focused on the most prominent inducers of EMT such as the zinc finger E-box binding homeobox (ZEB1 and ZEB2), the zinc-finger transcriptional repressor (Snail and Slug), and the basic helix-loop-helix transcription factor (Twist1) through searching the published literature [6, 7], knowing that EMT-TFs are directly or indirectly involved in metastasis of malignant cells through a series of signaling cascades, including the wingless-related integration site(Wnt), serine/threonine-specific protein kinase (Akt), mitogen-activated protein kinase (MAPK) and signal transducer and activator of transcription 3 (STAT3) pathways [8, 9].

During the past decade, much research has begun noticing the correlation between the expression of EMT-TFs and the prognosis of HCC. However, the results are often unconvincing due to the limited sample sizes. Here, we sought to perform a meta-analysis to evaluate clinicopathological and prognostic significance of EMT-TFs overexpression in HCC patients, especially those with high incidences of recurrence after curative resection.

## RESULTS

### Study selection and patient characteristics

**Figure 1:**
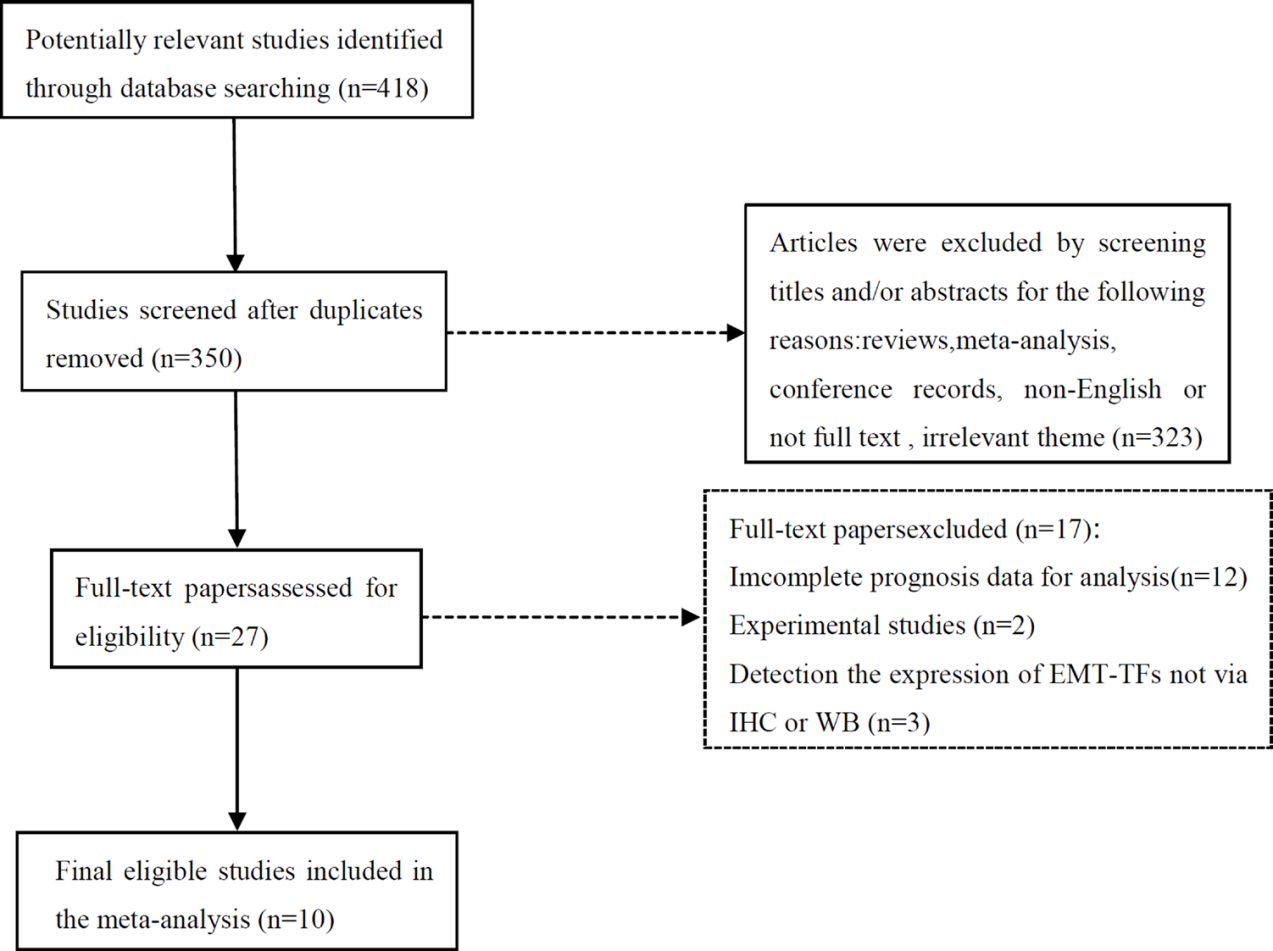
Flow chart of literature selection for the meta-analysis

The initial search identified 418 potentially relevant studies. After screening, 10 published studies including 1,334 patients were selected for this pooled analysis [10–19]. A flowchart depicting the selection of the eligible literature is shown in Figure 1.

All the included studies were retrospectively analyzed, with the sample size ranging from 40 to 323 (median 133). The overexpression of EMT-TFs was reported in 662 (49.6%) of the 1,334 included patients. The highest positive expression rate was Twist1, accounting for 60.3%, followed by Snail (51.9%), ZEB2 (50.3%), ZEB1 (43.6%) and Slug (29.4%). These studies were published between 2007 and 2015. Among all cohorts, Asia was the only source region of the 10 included studies, including 9 studies from China [11–19] and one from Japan [10]. Newcastle-Ottawa Quality Assessment Scale was applied to assess these studies. The result showed that the quality scores ranged from 5 to 8 (median 6.5), indicating a relatively high study quality.

Characteristics of the included studies are listed in Table 1. All the studies focused on OS, with a median follow-up period of at least 48 (48–80) months. The definition of EMT-TFs positive expression was based on immunohistochemistry (IHC) or western blot analysis (WB) evaluation in all the eligible articles, as expressed as the percentage of positive cells or/and staining intensity. Hazard ratios (HRs) and 95% confidence intervals (CIs) were directly recorded in 8 studies [10–12, 15–19] and could be inferred from two other studies using the Tierney's methods described above, among which one [14] were calculated by variance and *P* value, and the other [13] was estimated only by Kaplan-Meier survival curves.

**Table 1:** Characteristics of the included studies

EMT-TFs	Author	Year	Country	Case	EMT-TFs Positive(%)	Treatment	Antibody				method	Outcome	MFu time (months)	NOS score
							source	type	dilution	company				
ZEB1	Motoyuki	2013	Japan	108	23 (21.3)	Surgery	goat	polyclonal	1:100	SantaCruz, CA, USA	IHC	OS	60	8
	Zhou	2011	China	110	72 (65.5)	Surgery	NA	NA	NA	SantaCruz, CA, USA	WB	OS	60	7
ZEB2	Cai	2012	China	248	150 (60.5)	Surgery	rabbit	polyclonal	1:100	Sigma, St.Louis, USA	IHC	OS	80	8
	Yang	2015	China	92	21 (22.8)	Surgery	rabbit	polyclonal	1:100	Abcam, Cambridge, UK	IHC	OS	60	5
Snail1	Zhou	2014	China	323	161 (49.8)	Surgery	NA	NA	NA	Novus, USA	IHC	OS	60	6
	Zhao	2012	China	97	57 (58.8)	Surgery	NA	NA	1:250	SantaCruz, CA, USA	IHC	OS	60	7
Slug	Zhang	2013	China	119	35 (29.4)	Surgery	NA	NA	NA	Danvers, MA, USA	IHC	OS	60	8
Twist1	Zhang	2010	China	100	70 (70.0)	Surgery	rabbit	polyclonal	1:50	SantaCruz, CA, USA	IHC	OS	76	7
	Zhao	2011	China	97	51 (52.6)	Surgery	NA	NA	1:100	SantaCruz, CA, USA	IHC	OS	60	7
	Niu	2007	China	40	22 (55.0)	Surgery	rabbit	monoclonal	1:50	SantaCruz, CA, USA	IHC	OS	48	6

Abbreviations: EMT-TFs = epithelial to mesenchymal transition-inducing transcription factors; NA = not available;

IHC = Immunohistochemistry; WB = Western Blot; MFu = median Follow-up; OS = overall survival; NOS = Newcastle-Ottawa Scale.

### Evidence synthesis

### EMT-TFs and OS in HCC

**Figure 2:**
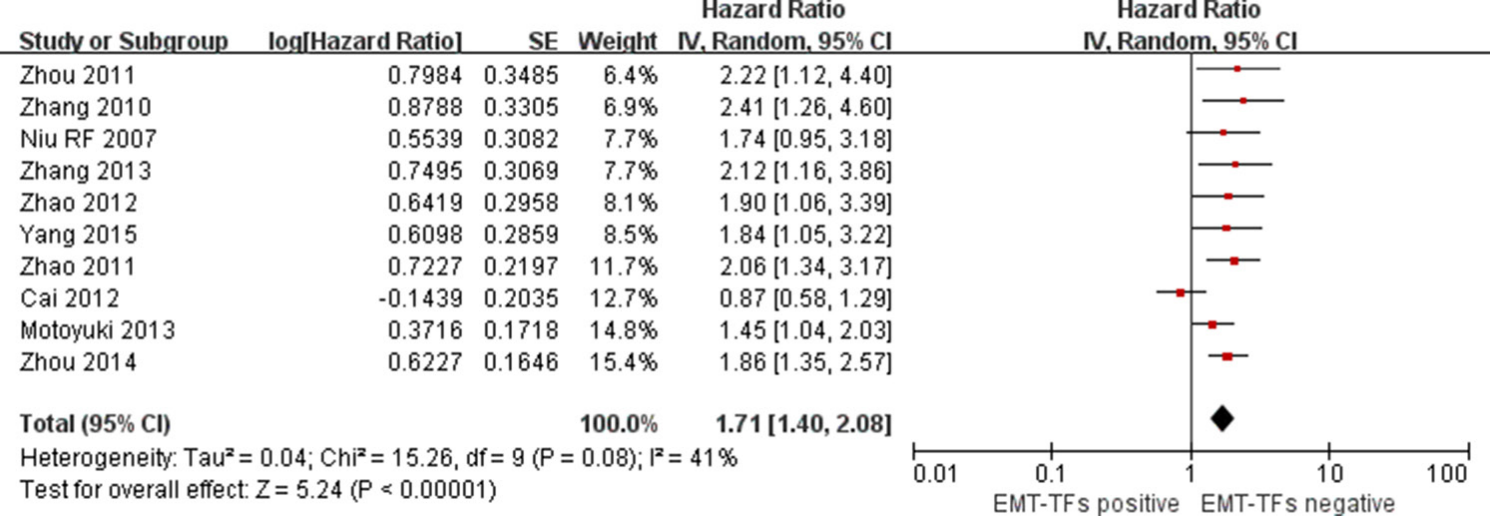
Forest plot of comparison between EMT-TF overexpression and EMT-TFs low/negative expression on OS in HCC patients

The pooled HR for OS indicated that EMT-TF positive expression was associated with poor OS [HR = 1.71; 95% CI: 1.40–2.08; *p* < 0.00001] in HCC with a statistically significant 71% increase in the risk for mortality (Figure 2). Seeing that the heterogeneity test showed a *P* value of 0.08 and an I^2^ statistic index of 41%, we considered that there may be relatively substantial heterogeneity between these studies, and therefore we used the random-effects model.

**Figure 3:**
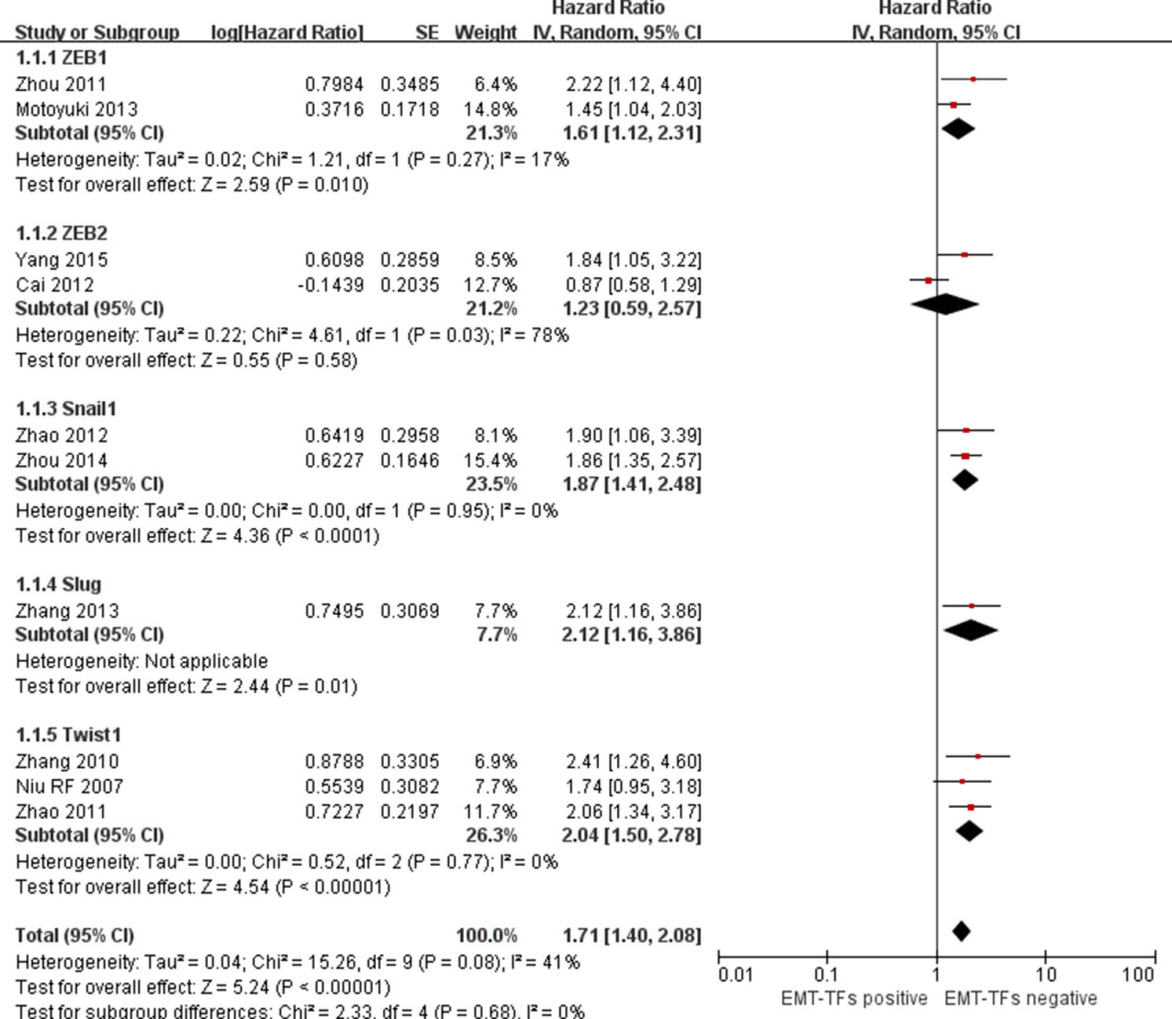
Forest plot describing subgroup analysis of the association between individual EMT-TF overexpression and OS in HCC patients

Figure 3 shows the impact of various individual EMT-TFs on the survival of HCC patients. The significantly higher HRs for OS was Slug [HR = 2.12; 95% CI: 1.16–3.86; *p* = 0.01]. But as the transcription factor was reported in only one study, the result should be considered with caution. In addition to ZEB2 [HR = 1.23; 95% CI: 0.59–2.57; *p* = 0.58], HRs for Twist1 [HR = 2.04; 95% CI: 1.50–2.78; *p* < 0.00001], Snail1 [HR = 1.87; 95% CI: 1.41–2.48; *p* < 0.0001], and ZEB1 [HR = 1.61; 95% CI: 1.12–2.31; *p* = 0.01] suggested that their positive expression correlated with poor OS.

### EMT-TFs and clinicopathological features in HCC

In the meta-analysis, the pooled data revealed that the associations between EMT-TFs and the following clinicopathological features were significant: TNM stage [III+IV vs. I+II; OR = 2.18; 95% CI: 1.08–4.38; *p* = 0.03], histological differentiation [poor vs. well+moderate; OR = 1.96; 95% CI: 1.22–3.17; *p* = 0.006], intrahepatic metastasis [pos vs. neg; OR = 2.94; 95% CI: 1.56–5.54; *p* = 0.0009] and vascular invasion [pos vs. neg; OR = 3.09; 95% CI: 1.67–5.73; *p* = 0.0003]. Therefore, the findings from the subgroup analysis were consistent with the conclusion that EMT-TFs as a poor prognostic factor. However, no significant association between EMT-TFs overexpression and age (> 55 vs. ≤ 55), gender (male vs. female), tumor size (> 5 cm vs. ≤ 5 cm), cirrhosis (yes vs. no), hepatitis B surface antigen (pos vs. neg) or AFP (> 20 ng/ml vs. ≤ 20 ng/ml) was found. The details of the subgroup analysis results are summarized in Table 2.

**Table 2:** Correlation of EMT-TFs overexpression and clinicopathological features in HCC

variable	No.of	No.of	OR (95% CI)	*P*	Heterogeneity		Model used
	studies	patients			*I*^2^ (%)	Ph	
TNM stage (III+IV vs. I+II)	6	766	2.18 (1.08–4.38)	0.03	76	0.0008	random
Differentiation (poor vs. well+ moderate)	5	435	1.96 (1.22–3.17)	0.006	0	0.58	fixed
Intrahepatic metastasis (pos vs. neg)	3	258	2.94 (1.56–5.54)	0.0009	0	0.86	fixed
Vascular invasion (pos vs. neg)	2	218	3.09 (1.67–5.73)	0.0003	0	0.98	fixed
Age (> 55 vs. ≤ 55)	2	219	1.27 (0.71–2.26)	0.42	48	0.16	fixed
Gender (male vs. female)	7	817	1.32 (0.89–1.96)	0.17	0	0.80	fixed
Tumor size (> 5 cm vs. ≤ 5 cm)	6	687	1.09 (0.62–1.91)	0.76	63	0.02	random
Cirrhosis (yes vs. no)	3	458	0.83 (0.55–1.25)	0.38	50	0.13	fixed
HBSAg (pos vs. neg)	4	498	1.30 (0.75–2.26)	0.35	0	0.89	fixed
AFP (> 20 ng/ml vs. ≤ 20 ng/ml )	4	483	0.90 (0.42–1.96)	0.80	68	0.02	random

Abbreviations: HBSAg = hepatitis B surface antigen; AFP = alpha fetoprotein; pos = positive; neg = negative.

### Assessment of possible publication bias and sensitivity analysis

**Figure 4:**
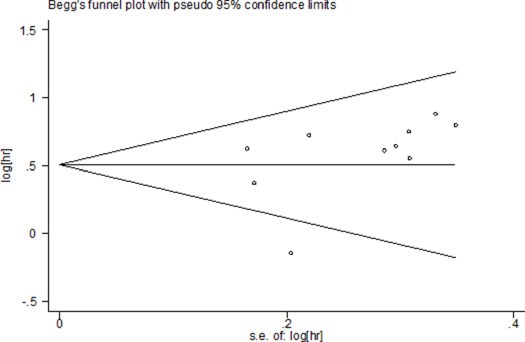
Publication bias using Begg's funnel plots for OS

The possible publication bias among these eligible studies was evaluated by applying the Begg's funnel plot and the Egger's test. As illustrated in Figure 4, visual assessment of the funnel plots shapes revealed no obvious publication bias for OS, and evaluation using Egger's test also failed to discover solid evidence for significant publication bias (*t* = 1.33; *p* = 0.220, Figure not shown). When the number of studies was smaller than 10, publication bias was not investigated because of the low sensitivity of the quantitative and qualitative tests [20]. In such cases, we performed the sensitivity analysis by removing one study at each time. The result demonstrated that not a single study had remarkable impact on the overall HRs. Thus, the above results further verified that the general conclusions of this current meta-analysis were credible.

## DISCUSSION

Early diagnostic and surgical techniques of HCC have been improved greatly within the past decade. However, recurrence and metastasis remains one of the major threats and the most critical aspect of HCC, because it is the key event causing most cancer-related deaths [1, 21]. EMT is considered to be one of the key initial steps in cancer development, progression and metastasis, knowing that EMT can induce dissemination of malignant cells, thereby increasing cell migration and invasion [22, 23]. The concept of EMT and its reverse process, mesenchymal to epithelial transition (MET), was first recognized in the field of embryology, and is now known to play diverse roles in embryonic development and a series of physiological processes such as gastrulation, neural tube formation, tissue homeostasis, wound healing, stem cell plasticity, and organ fibrosis [24, 25]. There are growing studies reporting that EMT is involved not only in tumor metastasis and progression but in cancer recurrence and resistance to conventional adjuvant therapies [26, 27].

Recent studies found that EMT-TFs were overexpressed in cancer patients, suggesting that numerous EMT-inducing transcription factors may act as primary molecular switches to induce the EMT process by activating or inhibiting the known signaling pathways [8, 28]. A meta-analysis of 3218 patients from 14 studies was published in 2016 demonstrated that the overexpression of EMT-TFs was a poor prognostic factor for metastatic breast cancer [HR = 1.72; 95% CI: 1.53–1.93; *p* = 0.001] [29]. Accordingly, there has been great interest to confirm whether EMT-TFs high expression could be used as a potential prognostic biomarker for HCC to help guide surveillance and clinical decision-making regarding adjunctive therapies. However, there is no comprehensive analysis to draw a generally accepted conclusion.

In the current meta-analysis, we collected all data available from published articles to assess the correlation between EMT-TFs expression and HCC prognosis after resection for the first time. The pooled HR results suggest that the up-regulated expression of EMT-TFs (ZEB1, Snail, Slug, and Twist1) may contribute to the adverse prognosis of HCC. In addition, our study also indicates the predictive value of EMT-TFs high expression for HCC metastasis and progression.

According to the results of evidence synthesis, we consider EMT-TFs high expression as a new biomarker and a risk factor for the prediction of the HCC outcome after resection. There are some possible explanations for the close association of EMT-TF high expression with poor prognosis in HCC. First of all, EMT-TFs together with other factors can specifically bind to the E-box DNA sequences within the E-cadherin promoter, recruit transcriptional corepressors and histone deacetylases, thereby repressing E-cadherin expression and acquiring the expression of mesenchymal markers, such as N-cadherin, Vimentin, and α–SMA [30, 31]. Afterwards, it regulates the EMT process directly or indirectly by activating or inactivating the known signaling pathways. Second, recent evidence has supported the discovery that EMT-TFs overexpression is closely linked to the induction of cancer stem cell (CSC) phenotype that possesses self-renewal properties in various types of human cancers, thus enhancing tumorigenesis and helping resistance to chemo/radiation therapy associated with CSC characteristics [32–34]. Third, the up-regulated expression of EMT-TFs induces tumor invasion and metastasis. For instance, Snail was found to induce cancer cell invasion through regulating the expression of MMP proteins in HCC [35]. In addition, EMT-TFs also can regulate angiogenic factors and hypoxia-inducible factor-1 alpha (HIF-1α) to promote tumor angiogenesis in HCC [36]. Finally, several studies have also suggested that EMT-TFs play a critical role in the regulation of anti-apoptosis and anti-cancer drug resistance [37, 38].

Consequently, EMT, CSC generation, tumor invasion and metastasis, and angiogenesis are closely associated with the transformation of cancer cells to more aggressive behavior. These roles of EMT-TFs may help partially explain why HCC patients with EMT-TF overexpression had significantly shorter OS than those with EMT-TF low expression.

During EMT, tumor cells gradually lose the epithelial markers (E-cadherin, tight junction protein-1, laminin and cytokeratin) and obtain the expression of mesenchymal markers (N-cadherin, Vimentin and α–SMA). Among them, one of the essential hallmarks of EMT is the loss of E-cadherin function, which is really important to adequately understand the whole regulation mechanism of EMT-TFs as the upstream molecules of E-cadherin [39]. A variety of signaling pathways are triggered by EMT-TFs, including the Akt, MAPK, STAT3, transforming growth factor beta (TGFβ), β-catenin, Wnt, Ras, and Notch pathways. In addition to the classical triggering signaling pathways, some signaling molecules such as epidermal growth factor (EGF), nuclear factor kappa B (NF-κB), fibroblast growth factor (FGF), neurotrophic receptor tyrosine kinase B (Tr-kB), hepatocyte growth factor (HGF), steroid receptor co-activator (SRC)-3 protein, necrosis factor alpha (TNF-α), and HIF-1α are all activated [8, 9]. The coordination of these factors results in the repression of E-cadherin expression. Thus, success in targeting EMT-TFs via RNA interference (RNAi) technology or specific chemotherapeutic drugs will provide a new approach for the control of cancer metastasis.

There are several limitations in this meta-analysis, even though efforts have been made to comprehensively evaluate clinicopathological and prognostic significance of EMT-TFs overexpression in HCC. First, different antibodies, dilution solubility and cut-off values will impact the accuracy of assessment that the positive expression of EMT-TFs. Hence, a large multicenter clinical study using the same antibody and cut-off values may be helpful to gain more credible results. Second, there may be potential language bias in this meta-analysis, because the search strategy was limited to studies published in English only. In addition, the eligible articles included only Asian populations, thus lacking the homogeneity of the population distribution. Third, not all the studies directly provided HRs and 95% CIs, so the data extracted by using Tierney's methods may also cause the imprecision of the original data.

Despite these limitations, the results of our meta-analysis initially support the hypothesis that EMT-TF overexpression is associated with malignant phenotype features and poor postoperative OS of HCC patients in Asian populations. More investigations are needed in order to fully understand the pivotal role of each individual EMT-TF so as to provide new insights into tumor metastasis and progression, and lay a theoretical foundation for innovating target-specific drug therapies and molecular prognostic biomarkers of HCC after resection.

## MATERIALS AND METHODS

### Literature search strategy

A comprehensive systematic literature search in the PubMed, Web of Science database and Cochrane Library was performed to retrieve all the relevant articles (deadline until December 31, 2016 ), with the limit to “human” and papers published in English. The initial electronic search strategies included using the random combination of following Medical Subject Heading (MeSH) search terms: “ZEB, Snail, Slug or Twist1 ”, “hepatocellular carcinoma”, and “prognosis”. In addition, reference lists from identified primary articles were then once again manual cross-searched to identify any studies that were omitted by the search strategies. In the situation when multiple studies overlapped patient cohorts, only the published research with the largest sample size was included in the analysis.

### Data extraction

The titles and abstracts of all candidate articles were read independently by two reviewers (TW. and TZ.), and irrelevant ones were subsequently excluded according to the PICO principle [40]. Then, articles that could not be classified based on the abstracts alone were required for full-text scrutinization. Finally, eligible studies were carefully selected according to the following inclusion criteria. If any disagreement or discrepancy occurred in the eligibility of studies, the two reviewers would conduct a debate or consult the third reviewer (YZ.) until a consensus was reached. Quality assessment was conducted for each of the acceptable studies by two reviewers independently (YZ. and TZ.) using the Newcastle–Ottawa Quality Assessment Scale (NOS) [41]. Parameters were extracted from each included paper, including the first author's name, publication year, country, number of total patients, cases with positive expression rates of EMT-TFs, TNM stage, follow-up period, HRs and 95% CIs and *P*-values for OS. OS was defined as the period from the time of confirmed diagnosis of HCC to death, regardless of the patients receiving treatment or not. If the HRs were not directly shown in the article, we tried to contact the authors for additional data. If the authors did not reply, we extracted data from Kaplan-Meier survival curves by applied the Engauge Digitizer V4.1, and then the Tierney's methods was utilized to calculate the HRs and 95% CIs [42].

### Criteria for inclusion and exclusion

To be eligible for selection of this meta-analysis, studies were required to fulfill the following criteria: (1) patients were histologically confirmed as HCC; (2) the expression of EMT-TFs (ZEB1, ZEB2, Snail, Slug, Twist1) was measured by IHC or WB; (3) studies provided the correlation between EMT-TFs and OS; (4) studies reported HRs with 95% CIs, or calculation of these statistics from the data and survival curves presented; and (5) articles were published as papers in English.

Letters, reviews, editorials, abstracts, expert opinions, experiments that were performed on cell lines or animals, and articles that had inadequate original survival data for further analysis were excluded from this meta-analysis

### Statistical analysis

All the statistical analyses were performed via Review Manager 5.3 (The Cochrane Collaboration, Oxford, UK) and Stata 12 (Stata Corporation, College Station, TX, USA) in the meta-analysis. For the pooled analysis of the correlation between EMT-TFs expression and the clinical prognosis, HRs and 95% CIs for OS were combined to calculate the effective value (logHR and SE). As for the impact of EMT-TFs on clinicopathologic parameters of HCC, the pooled ORs and 95% CIs were used. Statistical heterogeneity was evaluated through the chi-squared test and *I*^2^ test. A chi-squared *P* value < 0.10 indicated the presence of statistically significant heterogeneity [43]. Pooled effects were calculated using either a fixed-effect or random-effects model [44]. A pooled HR > 1 indicated a higher risk of poor survival. The potential publication bias was analyzed by the Egger's test and Begg's Funnel plots [45]. Sensitivity analysis was also tested by excluding each study individually. Two-tailed *P* values < 0.05 were considered to denote statistical significance.

## Abbreviations

HCC: hepatocellular carcinoma
EMT-TFs: epithelial to mesenchymal transition -inducing transcription factors
ZEB: zinc finger E-box binding homeobox
SNAI: zinc-finger transcriptional repressor
Twist: basic helix-loop-helix transcription factor
OR: odds ratios
HR: hazard ratios
OS: overall survival
CI: 95% confidence interval
IHC: immunohistochemistry
WB: western blot analysis
CSC: cancer stem cell
MeSH: Medical Subject Heading
NOS: Newcastle–Ottawa Scale
